# A Sn-stabilized Cu^*δ*+^ electrocatalyst toward highly selective CO_2_-to-CO in a wide potential range†

**DOI:** 10.1039/d2sc04607e

**Published:** 2022-09-26

**Authors:** Xingxing Tan, Weiwei Guo, Shoujie Liu, Shunhan Jia, Liang Xu, Jiaqi Feng, Xupeng Yan, Chunjun Chen, Qinggong Zhu, Xiaofu Sun, Buxing Han

**Affiliations:** Beijing National Laboratory for Molecular Sciences, CAS Key Laboratory of Colloid and Interface and Thermodynamics, CAS Research/ Education Center for Excellence in Molecular Sciences, Institute of Chemistry, Chinese Academy of Sciences Beijing 100190 P. R. China sunxiaofu@iccas.ac.cn hanbx@iccas.ac.cn; School of Chemical Sciences, University of Chinese Academy of Sciences Beijing 100049 P. R. China; College of Chemistry and Chemical Engineering, Qingdao University Qingdao 266071 P. R. China; Chemistry and Chemical Engineering of Guangdong Laboratory Shantou 515063 P. R. China; Shanghai Key Laboratory of Green Chemistry and Chemical Processes, School of Chemistry and Molecular Engineering, East China Normal University Shanghai 200062 P. R. China

## Abstract

Current techno-economic evaluation manifests that the electrochemical CO_2_ reduction reaction (eCO_2_RR) to CO is very promising considering its simple two-electron transfer process, minimum cost of electricity, and low separation cost. Herein, we report a Sn-modification strategy that can tune the local electronic structure of Cu with an appropriate valence. The as-prepared catalysts can alter the broad product distribution of Cu-based eCO_2_RR to predominantly generate CO. CO faradaic efficiency (FE) remained above 96% in the wide potential range of −0.5 to −0.9 V *vs.* the reversible hydrogen electrode (RHE) with CO partial current density up to 265 mA cm^−2^. The catalyst also had remarkable stability. Operando experiments and density functional theory calculations demonstrated that the surface Cu^*δ*+^ sites could be modulated and stabilized after introducing Sn. The Cu^*δ*+^ sites with low positive valence were conducive to regulating the binding energy of intermediates and resulted in high CO selectivity and maintained the stability of the catalyst. Additionally, scaling up the catalyst into a membrane electrode assemble system (MEA) could achieve a high overall current of 1.3 A with exclusive and stable CO generation.

## Abstract 2

Sn-stabilized Cu^*δ*+^ electrocatalysts have been prepared, achieving highly selective CO_2_-to-CO in a wide potential range. The modifier Sn could tune the electronic structure of Cu, resulting in high reactivity and selectivity for CO production.

## Graphical abstract

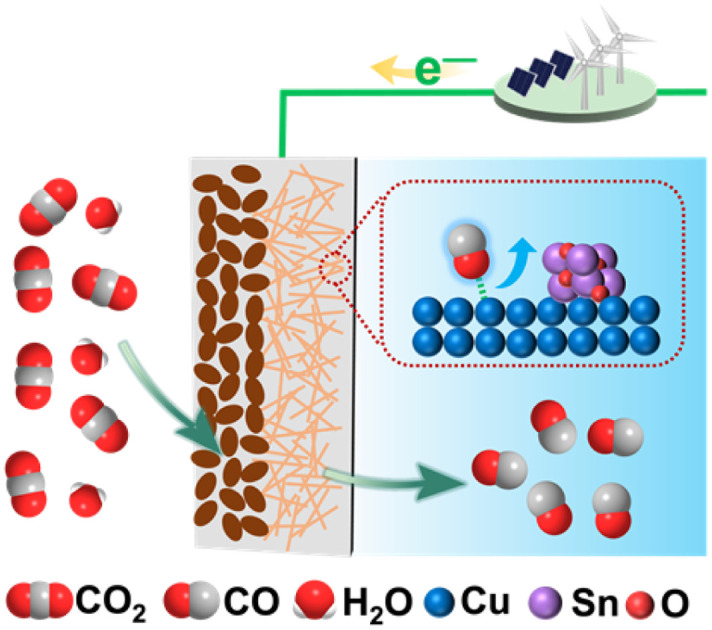

## Introduction

The electrochemical CO_2_ reduction reaction (eCO_2_RR) to value-added chemicals and fuels provides a sustainable route for storing renewable electrical energy and closing the carbon loop.^1–5^ CO_2_ can be converted to various products *via* different H^+^/e^−^ transfer numbers and reduction pathways. Among them, CO is a component for syngas, which has been widely used in the chemical synthesis of a range of organic products such as alcohol, olefins, and aromatics based on the Fischer–Tropsch route.^6,7^ The eCO_2_RR is a potential substitute technology for CO production due to the high cost and high energy consumption of the traditional methods (*e.g.* reverse water gas shift reaction and natural gas reformation).^8–10^ Despite recent improvements on exploiting various electrocatalysts and electrolytes,^11–14^ the scaling up of the eCO_2_RR to CO for practical applications is still in its infancy with a few urgent issues to be solved such as high catalyst cost, high overpotential, narrow potential window, low current density, as well as poor long-term stability.

The 2H^+^/2e^−^ reaction process of CO_2_-to-CO involves multiple steps, including the formation of *COOH, the dissociation of *COOH to *CO and CO desorption.^15,16^ Thus, an ideal catalyst for CO_2_-to-CO should have appropriate adsorption strengths for *COOH and *CO. As an abundant, cheap, and less toxic element, Cu is a good candidate for the substitution of noble metals in practical application.^17,18^ A Cu-based catalyst is also the most promising catalyst for the eCO_2_RR, and surface Cu^*δ*+^ (0 < *δ* < 1) sites have been suggested as active sites.^19–21^ However, a Cu catalyst is capable of converting CO_2_ to a range of reduced products (CO, hydrocarbons, and oxygenates),^10,22^ giving rise to poor selectivity for a specific product, especially for CO. Moreover, it suffers from low yields and catalyst durability issues under electrochemical conditions.^23^ Therefore, there is a strong need for developing an efficient strategy to optimize the intermediate binding strength on Cu^*δ*+^ sites and reduce the tendency of Cu^*δ*+^ reduction at negative potentials.

Introducing modifier elements into Cu has been shown to be a promising route to achieve high selectivity for the eCO_2_RR. Prior studies showed that some non-metallic elements (*e.g.*, boron and halides)^19,22^ and metals (*e.g.*, Au and Pd)^24–26^ can tune the local electronic structure of Cu with an appropriate valence to meet the demands for diverse products. p-Block metals (pM), such as In, Sn, and Pb, possess O affinity and high overpotential for the H_2_ evolution reaction (HER).^27–30^ Doping pM into Cu may prefer to bond with O in *COOH,^31^ which benefits *COOH dissociation to form *CO. Moreover, it can also suppress the undesirable HER. On the other hand, the gas diffusion electrode (GDE) has shorter gas diffusional lengths and higher concentrations of gaseous feeds,^12,32^ resulting in higher current densities. Thus, constructing an appropriate pM-Cu GDE may realize high selectivity and activity for CO_2_-to-CO.

Herein, we have constructed a Sn-modified Cu electrocatalyst that can significantly enhance the CO_2_-to-CO activity and selectivity with high FE over a wide potential range. Experimental and density-functional theory (DFT) studies indicated that the introduction of Sn could modulate the local electronic structure of the Cu-based catalyst and stabilize the oxidation states of Cu during the eCO_2_RR process. Cu^0^ was replaced by Cu^*δ*+^ sites with appropriate valence during the eCO_2_RR after introducing Sn. The resulting Cu^*δ*+^ sites could facilitate CO_2_ activation and CO formation. Besides, scaling up the electrode into a modular cell could achieve a very high current with exclusive and stable CO generation.

## Results and discussion

### eCO_2_RR performance in a flow cell

Sn-modified Cu catalysts were fabricated by a facile coprecipitation method followed by annealing at 600 °C for 2 h under an argon atmosphere. The obtained catalysts had Sn contents of 0, 0.6, 2.9, and 4.0 wt%, which were determined by inductively coupled plasma optical emission spectroscopy (ICP-OES). We denoted them as Cu–O, 0.6%Sn–Cu–O, 2.9%Sn–Cu–O, and 4.0%Sn–Cu–O for clarity. The electrocatalytic CO_2_ reduction performances were evaluated in a flow cell with 1 M KOH aqueous solution as electrolyte. The as-prepared catalyst inks were air-brushed on carbon paper with a hydrophobic microporous gas diffusion layer, which was used as the GDE.

**Fig. 1:**
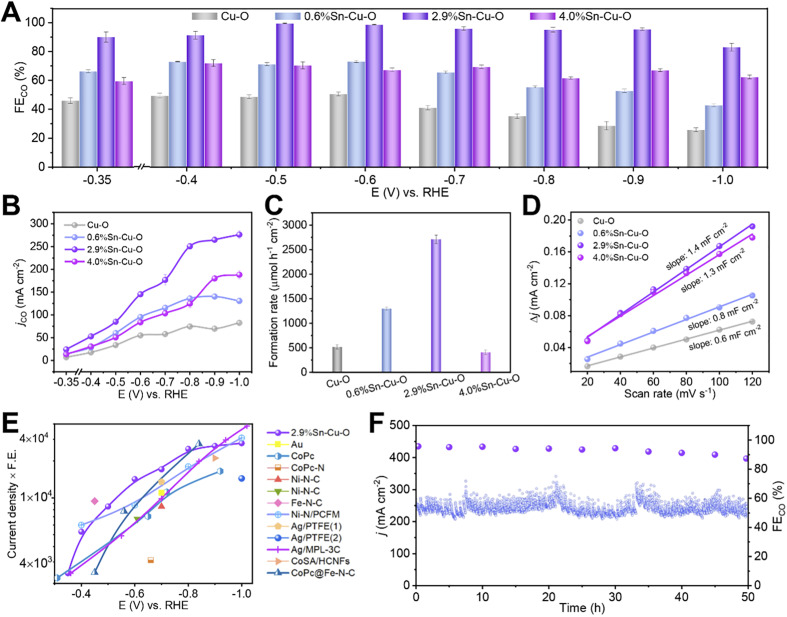
Electrocatalytic CO_2_RR performances. (A) The FE_CO_ and (B) *j*_CO_ at different applied potentials over Cu–O, 0.6%Sn–Cu–O, 2.9%Sn–Cu–O, and 4.0%Sn–Cu–O catalysts. (C) The formation rates of the CO product at the potentials with optimum CO selectivity over Cu–O, 0.6%Sn–Cu–O, 2.9%Sn–Cu–O, and 4.0%Sn–Cu–O catalysts. (D) The charging current density differences plotted against scan rates. (E) Overview of different catalysts' performance in flow cells reported so far (see Table S2† for details). (1) and (2) in (E) represent the electrocatalytic reactions conducted in KHCO_3_ and KOH, respectively. (F) The stability of 2.9%Sn–Cu–O obtained at −0.8 V.

The gas and liquid products were quantified by using gas chromatography (GC) and ^1^H nuclear magnetic resonance (NMR) spectroscopy, respectively. Cu–O showed a poor product selectivity with a mixture of H_2_, CO, formate, and C_2_ products (Fig. 1A and S1†). Doping Sn into CuO significantly improved the performance for CO_2_ reduction to CO. All the catalysts presented a combined product FE of around 100% in the potential range from −0.35 to −1.0 V *versus* RHE. It can be clearly observed that the addition of Sn could boost the activity and selectivity for CO formation, and 2.9%Sn–Cu–O exhibited the best performance (Fig. 1A, B and S2†). The change in applied potential from −0.35 V to −0.9 V over 2.9%Sn–Cu–O led to a variation in CO partial current density in a broad range from 24 to 265 mA cm^−2^, and the FE remained over 96% in the potential range of 0.5 to 0.9 V *vs.* RHE. This wide potential window is essential for expanding the eCO_2_RR process into practical applications, which is expected to enhance robustness and stability when inherent potential fluctuation occurs. However, as the Sn content was further increased up to 4.0 wt%, the main product was switched to formate, while the conversion of CO_2_ to CO was suppressed.

The formation rate of CO was also affected significantly by the Sn content. As presented in Fig. 1C, it can reach 2710 μmol h^−1^ cm^−2^ over 2.9%Sn–Cu–O at −0.6 V, which was roughly 5.2, 2.1 and 6.6 times higher than that of Cu–O, 0.6%Sn–Cu–O, and 4.0%Sn–Cu–O, respectively. We further normalized the CO formation rate based on the electrochemical surface area (ECSA), which was measured *via* the double-layer capacitance method (Fig. 1D and S3†). As a result, the normalized CO formation rate had the same sequence of 2.9%Sn–Cu–O > 0.6%Sn–Cu–O > Cu–O > 4.0%Sn–Cu–O at all the applied potentials (Fig. S4†).

The catalytic activity was then compared with that of the state-of-the-art catalysts by multiplying current density (activity) by CO formation FE (selectivity). It can be seen that the performance of 2.9%Sn–Cu–O was higher than many other reported catalysts at a lower overpotential (Fig. 1E, Tables S1 and S2†). Furthermore, the 2.9%Sn–Cu–O catalyst also displayed excellent stability and no obvious change in current density and FE was observed during the eCO_2_RR at −0.8 V for 50 h (Fig. 1F). Therefore, it is worth mentioning that the 2.9%Sn–Cu–O catalyst exhibited high selectivity for CO at a high current density in a wide operation window, which is very promising for application of the eCO_2_RR to CO.

### *Ex situ* characterization studies

**Fig. 2:**
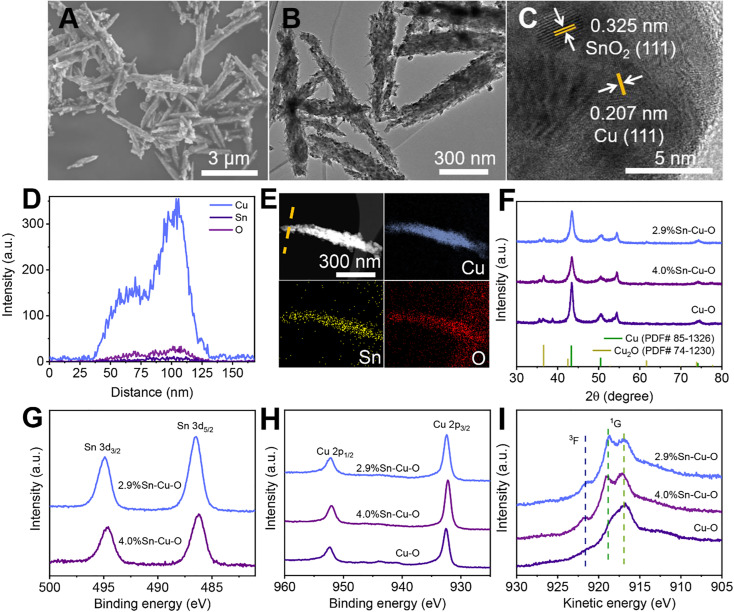
Morphology and structure characterization after the eCO_2_RR. (A) SEM image, (B) HR-TEM image, (C) HAADF-STEM image, (D) EDS line scan, and (E) EDS mapping of 2.9%Sn–Cu–O. (F) XRD patterns and quasi-operando X-ray photoelectron spectroscopy (XPS) of (G) Sn 3d, (H) Cu 2p, and (I) Cu L_3_M_45_M_45_ Auger spectra of Cu–O, 2.9%Sn–Cu–O, and 4.0%Sn–Cu–O catalysts.

The scanning electron microscopy (SEM), transmission electron microscopy (TEM), and powder X-ray diffraction (XRD) measurements clarified that Sn-modified CuO was formed with a rod-like structure after coprecipitating and annealing (Fig. S5–S9†). The actual catalyst was then formed *in situ* at the beginning of the electrochemical reduction, which will be further discussed in the following sections. The SEM and TEM images in Fig. 2A and B show that the morphology of 2.9%Sn–Cu–O remained almost unchanged with a length of 2.4 ± 0.6 μm and a diameter of 120 ± 40 nm. The high resolution TEM (HRTEM) image exhibited a lattice spacing of 0.207 nm and 0.325 nm, which can be ascribed to the Cu(111) and SnO_2_(110) planes,^33,34^ respectively (Fig. 2C). The line-scan analysis and element mapping analysis were then carried out, suggesting that Cu, Sn, and O elements dispersed uniformly over the catalyst (Fig. 2D and E). The Sn content measured by TEM energy-dispersive X-ray spectroscopy (EDS) for 2.9%Sn–Cu–O was 2.7 wt%, which was close to that obtained by ICP-OES. The TEM images of other catalysts after the reaction can be seen in Fig. S10† The XRD patterns in Fig. 2F showed that CuO was reduced *in situ* in the eCO_2_RR process. The diffraction peaks at 43.4°, 50.5°, 74.2°, and 36.5° can be assigned to the Cu(111), (200), (220), and Cu_2_O(111) planes, respectively. The characteristic peaks of SnO_2_ were not found due to its low loadings. The CO_2_ adsorption isotherms (Fig. S11†) showed that higher CO_2_ adsorption capability was acquired after introduction of Sn into CuO, while a similar CO_2_ adsorption capability was found in Sn-modified catalysts with different Sn contents. The difference in selectivity and activity may be attributed to the local electron structure of Cu and Sn in the as-prepared catalysts.

Quasi-*in situ* X-ray photoelectron spectroscopy (XPS) was performed to investigate the composition and valence state of the catalysts (Fig. S12†). As shown in Fig. 2G, the binding energy of 486.4 eV for Sn 3d_5/2_ was attributed to SnO_2_, which showed no obvious change in the Sn 3d region after the eCO_2_RR (Fig. 2G and S13†).^35^ XPS depth profiling analysis using an argon cluster beam and Ar^+^ to etch layers of the surface or surface contamination was performed to reveal subsurface information. As shown in Fig. S13C and D,† the XPS depth profiling analysis result exhibited a doublet with the main peak for the Sn 3d_5/2_ component at a binding energy 486.5 eV after the eCO_2_RR, which was in agreement with the value for SnO_2_. No new peaks of Sn^2+^ and Sn^0^ can be found. There was also no obvious change in the Sn 3d region after different times of Ar^+^ beam etching (the etching depth was estimated to be 30 to 380 nm). It indicated that the oxidation states of Sn did not undergo considerable change during electrocatalysis. Fig. S14 and S15† show that all the catalysts exhibited obvious Cu^2+^ features with strong satellite peaks at 943.0 eV and higher binding energies of Cu 2p_3/2_ at 933.7 eV before electrolysis.^36^ These results confirmed that both Cu and Sn in the matrix exist in their oxide states (Cu^2+^ and Sn^4+^) before electrolysis. However, the Cu 2p region for all the catalysts exhibited a shift toward lower binding energies after CO_2_ electrolysis (Fig. 2H and S14†), and the Cu Auger L_3_M_45_M_45_ transition spectra (^3^F and ^1^G) showed the valence configuration of Cu^+^ (with a ^1^G peak at 916.8 eV) and Cu^0^ (with a ^1^G peak at 918.8 eV and a distinct ^3^F peak) (Fig. 2I).^37,38^ The results revealed that CuO was reduced during the eCO_2_RR.

### Operando measurements

Operando X-ray absorption spectroscopy (XAS) was conducted to further investigate the detailed structural information and evolution process of catalysts during CO_2_ reduction (Fig. S16†). Similarly, the X-ray absorption near-edge structure (XANES) spectra at the Cu K-edge showed the typical features of the CuO reference (CuO-ref), indicating that Cu^2+^ was dominant in these three catalysts before the eCO_2_RR (Fig. S17†). As the cathodic potential was applied, the Cu oxides in the catalysts were reduced (Fig. S18†). Only features consistent with metallic Cu were detected in Cu–O, implying that Cu^2+^ was fully reduced to Cu^0^. However, the absorption edges of 2.9%Sn–Cu–O and 4.0%Sn–Cu–O resided between that of metallic Cu (Cu-ref) and the Cu_2_O reference (Cu_2_O-ref).

**Fig. 3:**
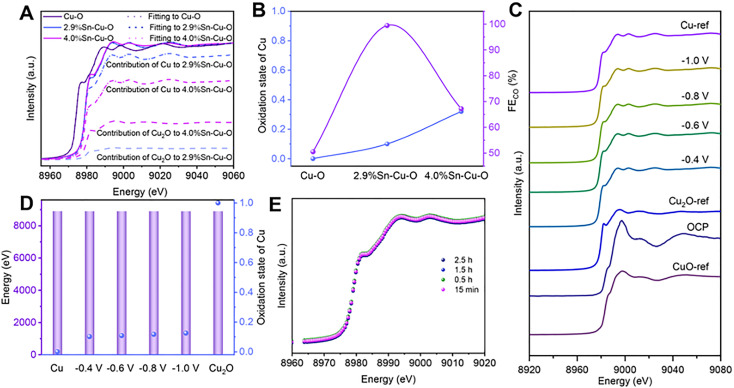
Operando XAS and XES of catalysts during the eCO_2_RR. (A) Cu K-edge XANES data fitting with a linear combination of reference spectra (at an applied potential of −0.6 V *vs.* RHE) for Cu–O, 2.9%Sn–Cu–O, and 4.0%Sn–Cu–O catalysts. (B) Evolution of FE_CO_ with the oxidation state of Cu at −0.6 V *vs.* RHE. (C) XANES spectra at the Cu K-edge of 2.9%Sn–Cu–O at different potentials (OCP: open circuit potential). (D) K*β*_1,3_ energies and the oxidation state of Cu in 2.9%Sn–Cu–O obtained by linear combination fitting of the XES data at different potentials. (E) Cu K-edge XANES spectra of 2.9%Sn–Cu–O measured at different times during the eCO_2_RR.

To estimate the Cu oxidation state more intuitively, a linear combination fitting analysis of the operando XANES data was performed using Cu foil and Cu_2_O as the reference spectra. The results suggested that the fraction of Cu^0^ species was increased from 10% to 27% as the Sn content varied from 2.9 to 4.0% (Fig. 3A and B), indicating that the introduction of Sn could modulate the oxidation state of Cu-based catalysts during the eCO_2_RR. This modulation effect likely stems from the moderate electronegativity of Sn^4+^ (1.73), which is between that of Cu^0^ (1.9) and Cu^+^ (1.56). Remarkably, it was found that the catalytic activity followed a volcano-shaped trend for the eCO_2_RR to CO with respect to the Cu oxidation state (Fig. 3B). The highest FE_CO_ of 99.5% was achieved at the Cu valence state of +0.1. We also estimated the Cu oxidation state as a function of the Cu K-edge energy shift and found that the Cu oxidation state in 2.9%Sn–Cu–O catalysts remained at around +0.1 at different cathodic potentials (Fig. 3C). Similar conclusions were also demonstrated by operando X-ray emission spectroscopy (XES), as the Cu K*β* lines are sensitive to the changes in the spin state and oxidation state. Similar K*β*_1,3_ energies were presented, implying that the oxidation state of Cu did not undergo considerable change at different cathode potentials (Fig. 3D). These results indicated the robustness of the oxidation state of Cu, which was attributed to the introduction of Sn.

Interestingly, similar FEs up to ∼90% were maintained in the catalytic activity toward CO_2_ reduction to CO at −0.4, −0.6, and −0.8 V *vs.* RHE, indicating that the stable Cu^*δ*+^ species (*δ* = 0.1) played a key role in the eCO_2_RR. A slight decrease in the FE_CO_ was observed at −1.0 V accompanied by an increase in FE of H_2_, which was attributed to the enhanced competitive HER at a high cathodic potential. The *in situ* Cu XANES spectra of 2.9%Sn–Cu–O catalyst at different time points were also recorded to observe the changes in the Cu oxidation state. As shown in Fig. 3E, similar Cu oxidation states of ∼+0.1 were observed after 15 min, 0.5 h, 1.5 h, and 2.5 h of operation at −0.6 V *vs.* RHE, implying that Cu^2+^ was reduced at beginning of electrolysis and the reduced Cu^*δ*+^ species were stable during the eCO_2_RR.

### DFT calculations

**Fig. 4:**
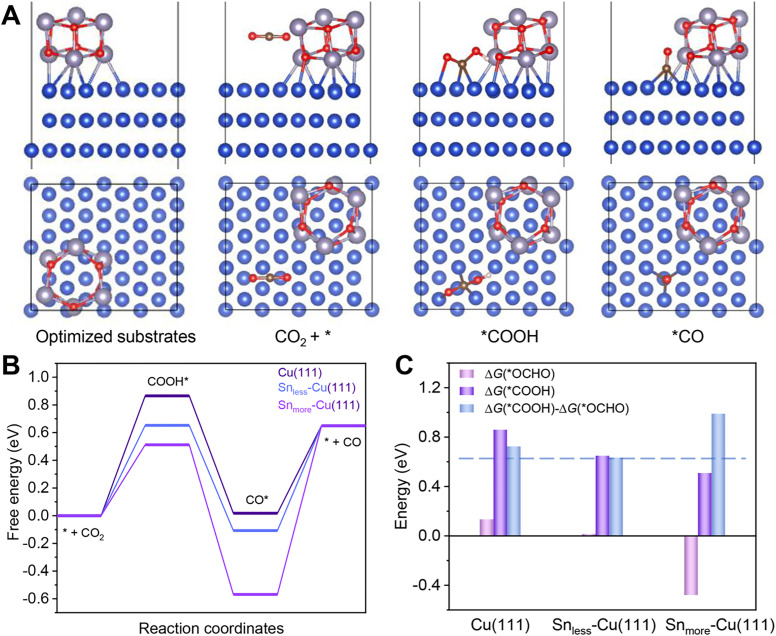
DFT calculations. (A) The catalytic pathway of CO_2_-to-CO on 2.9%Sn–Cu–O according to the optimized configurations with adsorbed intermediates. (B) Gibbs free–energy diagrams for the eCO_2_RR to CO on different simulated models. Blue, gray, red, brown, and white balls stand for Cu, Sn, oxygen, carbon, and hydrogen, respectively. (C) Adsorption energies of *COOH (G_*COOH_) and *OCHO (G_*OCHO_) over different models and their differences ((G_*COOH_)–(G_*OCHO_)).

DFT calculations were carried out to gain insight into the excellent performance of 2.9%Sn–Cu–O for CO_2_ reduction to CO. According to the HRTEM image and XRD patterns, we used the Cu(111) facet to represent Cu–O. Sn_less_–Cu(111) and Sn_more_–Cu(111) stand for 2.9%Sn–Cu–O and 4.0%Sn–Cu–O, respectively. All the computational structure models and the detailed data can be found in Fig. S20–S27 and Table S3.† The catalytic pathway of CO_2_-to-CO is illustrated in Fig. 4A. In general, *COOH and *CO are recognized as key intermediates in this 2H^+^/2e^−^ reaction process.^1,39^ The formation of *COOH on these models was endergonic, and the Gibbs free energy on Sn_less_–Cu(111) and Sn_more_–Cu(111) was 0.21 eV and 0.35 eV lower than that on Cu(111) (Fig. 4B). This indicated that the introduction of Sn enabled a decrease in the energy barrier of CO_2_ activation. Afterwards, the formation of *CO from *COOH was exergonic. Finally, CO desorption was highly endergonic, and acted as the rate-determining step (RDS) for Sn_less_–Cu(111) and Sn_more_–Cu(111). It was quite different from prisitine Cu, in which *COOH formation was the RDS. Based on the simulation, the Gibbs free energy of the RDS on Sn_less_–Cu(111) was 0.46 eV and 0.1 eV lower than that on Sn_more_–Cu(111) and Cu(111), respectively. It demonstrated a lower onset potential on Sn_less_-Cu(111) in the conversion of CO_2_-to-CO. Moreover, we also calculated the CO_2_-to-formate pathway on these three models. As shown in Fig. 4C and S27,† the formation of *OCHO on Sn_more_–Cu(111) was exergonic, and the difference of adsorption energy for *COOH and *OCHO followed the sequence of Sn_less_–Cu(111) < Cu(111) < Sn_more_–Cu(111), indicating the high selectivity for CO production on Sn_less_–Cu(111).

### eCO_2_RR using a membrane electrode assemble system (MEA)

**Fig. 5:**
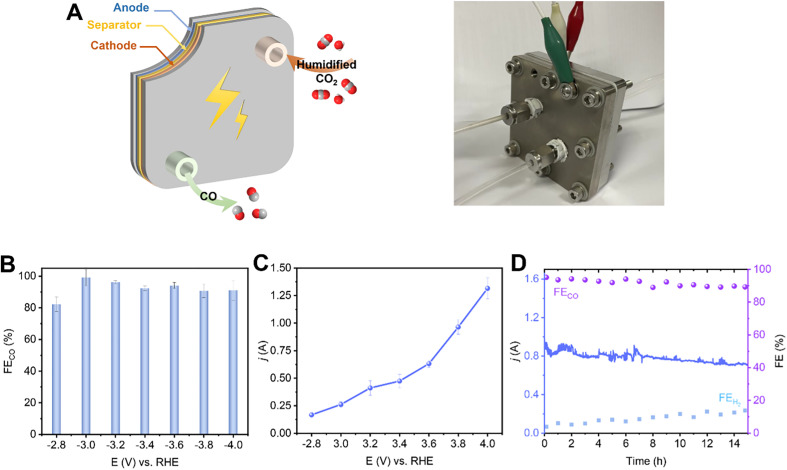
Scaling up the eCO_2_RR system over 2.9%Sn–Cu–O catalysts. (A) Photographs of the 6.25 cm^−2^-MEA system (B) The FE_CO_ at different applied potentials (−2.8 − −4.0 V, with no *iR* compensation). (C) The current–voltage response in the potential range of −2.8 to −4.0 V. (D) Stability test at a full-cell voltage of −3.8 V.

To evaluate the potential of the 2.9%Sn–Cu–O catalyst for practical applications, we integrated it into a 6.25 cm^−2^ membrane electrode assemble system. In this system, an anion exchange membrane (AEM) was sandwiched between the 2.9%Sn–Cu–O–GDE cathode and IrO_2_ anode to separate the chambers (Fig. 5A). The humidified CO_2_ gas was supplied to the cathode side with no catholyte and the anode side was circulated with 0.1 M KHCO_3_ anolyte. Fig. 5B shows potential-dependent FE_CO_ in the potential range of −2.8 to −4.0 V without *iR* compensation. The current–voltage response in the potential range of −2.8 to −4.0 V was recorded without *iR* compensation and is shown in Fig. 5C. The current increased with increasing voltage and a high eCO_2_RR current of −1.3 A was achieved at −4.0 V. The FE_CO_ increased concurrently, reaching the highest selectivity at −3.2 V with ∼96.2% FE_CO_. A high plateau of CO FEs about 90% was maintained across the potential range of −2.8 to −4.0 V (Fig. 5D). We finally assessed the stability of the 2.9%Sn–Cu–O electrode in an MEA system by potentiostatic electrolysis at a cell voltage of −3.8 V. It exhibited a steady eCO_2_RR current of about 0.8 A, along with continuous CO production with about 90% FE, throughout the 15 h electrolysis process.

## Conclusions

In summary, the Sn-modified Cu catalyst with stable Cu^*δ*+^ sites has been successfully prepared and applied for CO_2_ electroreduction. 2.9%Sn–Cu–O exhibited a high FE_CO_ over a wide potential range from −0.35 V to −1.0 V with remarkable stability. The outstanding performance originated from the modified electronic structure and the appropriate oxidation states of Cu. A detailed study suggested that Cu^*δ*+^ with a low valence state could regulate the binding energy of intermediates to give high CO selectivity. The modifier element Sn could maintain the stability of Cu^*δ*+^ sites during electrolysis, resulting in high reactivity and selectivity for CO production. The optimized catalyst was also applied in an MEA for large-scale production of CO with very high efficiency. This work presents a promising electrocatalyst for CO production approaching practical expectations. We believe that it may also inspire the design of new catalytic systems for other reaction pathways.

## Data availability

All experimental data is available in the ESI.†

## Author contributions

X. X. T. performed all the experiments. W. W. G., S. J. L., S.·H. J., L. X., J. Q. F, X. P.·Y., C.·C. J., and Q. G. Z. performed the analysis of experimental data. X. F. S. and B. X. H. co-supervised the whole project. All authors discussed the results and commented on the manuscript.

## Conflicts of interest

The authors declare no competing financial interests.

## Supplementary Material

SC-013-D2SC04607E-s001

SC-013-D2SC04607E-s002
