# Supplementary material for: A Sn-stabilized Cuδ+ electrocatalyst toward highly selective CO2-to-CO in a wide potential range
Source: Chem Sci. 2022 Sep 26;13(40):11918–25. doi: 10.1039/d2sc04607e (PMC9580469; doi:10.1039/d2sc04607e)
Supplement: SC-013-D2SC04607E-s001 [file SC-013-D2SC04607E-s001.pdf]

Electronic Supplementary Material (ESI) for Chemical Science.
This journal is © The Royal Society of Chemistry 2022

Electronic Supplementary Material (ESI) for Chemical Science. This journal is © The Royal Society of Chemistry 2022

Sn-Stabilized $Cu^{\delta+}$ Electrocatalyst toward Highly Selective CO_2-to-CO in a Wide Potential Range

Xingxing Tan,[1,2] Weiwei Guo,[1,2,3] Shoujie Liu,[4] Shunhan Jia,[1,2] Liang Xu,[1] Jiaqi Feng,[1] Xupeng Yan,[1,2] Chunjun Chen,[1] Qinggong Zhu,[1] Xiaofu Sun*[1,2] and Buxing Han*[1,2,5]

[1]Beijing National Laboratory for Molecular Sciences, CAS Key Laboratory of Colloid and Interface and Thermodynamics, CAS Research/ Education Center for Excellence in Molecular Sciences, Institute of Chemistry, Chinese Academy of Sciences, Beijing 100190, P. R. China

[2]School of Chemical Sciences, University of Chinese Academy of Sciences, Beijing 100049, P. R. China

[3]College of Chemistry and Chemical Engineering, Qingdao University, Qingdao 266071, P. R. China

[4]Chemistry and Chemical Engineering of Guangdong Laboratory, Shantou 515063, P. R. China

[5]Shanghai Key Laboratory of Green Chemistry and Chemical Processes, School of Chemistry and Molecular Engineering, East China Normal University Shanghai 200062, P. R. China

Experimental Section

Materials

Copper(II) chloride dihydrate ($CuCl_2 \cdot 2H_2O$, purity>99%), Stannic chloride pentahydrate ($SnCl_4 \cdot 5H_2O$, purity>99%), Sodium hydroxide (NaOH, purity>99%), potassium hydroxide (KOH, ≥ 85%), D_2O (98%), and sodium 2, 2-dimethyl-2-silapentane-5-sulfonate (DSS, 99%) were purchased from Alfa Aesar China Co., Ltd. Ethanol was obtained from Concord Technology (Tianjin) Co., Ltd. Citric acid (purity>99%) was purchased from Beijing Innochem Science & Technology Co. Ltd. Ar (99.999%) and CO_2 (99.999%) were provided by Beijing Analytical Instrument Company. Deionized water was used in the experiments. All the chemicals were used as received.

Preparation of catalyst samples

The 2.9%Sn-Cu-O catalyst was prepared by a facile coprecipitation method followed by annealing. First, 20 mL of $SnCl_4 \cdot 5H_2O$ (4.0 mmol) in ethanol was added into 140 mL of a mixed aqueous solution of $CuCl_2 \cdot 2H_2O$ (4.0 mmol) and citric acid (4.0 mmol) under vigorous stirring, followed by slow addition of 20 mL NaOH (2 M) aqueous solution with a constant stirring. After stirring continuously for 1 hour, 60 mL of NaOH (2 M) aqueous solution was added into the suspension and stirred for additional 30 min. Then, the precipitates were collected and washed three times with ethanol and deionized water. After centrifugation, the precipitates were dried under

vacuum at 60 °C for 12 h. The dried samples were annealed at 600 °C for 2 h under an argon atmosphere with a heating rate of 5 °C min^{-1}. The sample of Cu-O, 0.6%Sn-Cu-O, and 4.0%Sn-Cu-O were prepared by the same method, except that 0 mmol, 0.1 mmol, and 8.0 mmol $SnCl_4·5H_2O$ was added, respectively.

Characterization

The morphologies of the catalysts were characterized by a HITACHI SU8020 scanning electron microscope (SEM) and a JEOL JEM-2100F high-resolution transmission electron microscopy (HR-TEM). X-Ray diffraction (XRD) analysis of the samples was performed on a Rigaku D/max-2500 X-ray diffractometer with Cu-Kα radiation and the scan speed was 5° min^{-1}. X-ray photoelectron spectroscopy (XPS) analysis was performed on the Thermo Scientific ESCA Lab 250Xi using 200 W monochromatic Al Kα radiation. XPS depth profile spectra were collected on a Nexsa X-Ray Photoelectron Spectrometer (XPS) System (Thermo Fisher Scientific) using an argon cluster beam for 30 min (8 keV energy, 300 atoms size) and using Ar^+ for different times. The 500 μm X-ray spot was used. The base pressure in the analysis chamber was about 3×10^{-10} mbar. Typically, the hydrocarbon C1s line at 284.8 eV from adventitious carbon was used for energy referencing. The X-ray adsorption spectroscopy (XAS) measurements were performed using a modified flow cell at the 4B9A beamline at Beijing Synchrotron Radiation Facility (BSRF), China. The data were collected in fluorescence excitation mode using a Lytle detector. Cu foil, Cu_2O, and CuO were used as references. The acquired EXAFS data were

processed according to the standard procedures using the Athena and Artemis implemented in the IFEFFIT software packages. The XES data was performed at beamline 4W1B of the Beijing synchrotron Radiation Facility, China. The storage ring ran 2.5 GeV electron with current of 250 mA. A polychromatic beam (pink beam) with an incident X-ray energy of 5 or 10-18 keV was used, and the photon flux was on the order of 10^{13} phs/s. The beam spot-size (FWHM) was focused down to 50 μm by a polycapillary half-lens. The X-ray emission spectrum (XES) is performed at beamline 4W1B of the Beijing synchrotron Radiation Facility, China. The storage ring runs 2.5 GeV electron with current of 250 mA. A polychromatic beam (pink beam) with an incident X-ray energy of 10-18 keV is used, and the photon flux is on the order of 10^{13} phs/s. The beam spot-size (FWHM) is focused down to 50 μm by a polycapillary half-lens. The Kβ XES data are collected by means of a compact von Hamos spectrometer based on a full-cylinder Si crystal. The dispersion axis was perpendicular to the axis of the incident beam. The analyzers diffract and focus the emitted signal onto a position-sensitive detector. The 2D spectra were recorded using a Pilatus 100K detector with a pixel size of 172 × 172 μm^2. The XES raw data were processed by the standard procedures using the DAWN package.

Electrode preparation

To construct the cathode electrode, a catalyst slurry containing 5 mg of obtained catalysts, 1 mL of isopropanol and 20 μL of Nafion ionomer solution (5 wt% in H_2O) was first prepared and sonicated for 1 h. Then, the catalyst slurry (0.2 mL) was slowly

air-brushed onto the carbon paper with a hydrophobic microporous gas diffusion layer (YLS-30T GDL) under vacuum to achieve a catalyst loading of ~ 1.0 mg cm^{-2}. Ni foam were used as anode electrode.

Electrochemical study

Electrochemical reduction of CO_2 in a flow cell. Electrochemical studies were conducted in an electrochemical flow cell which was composed of a gas chamber, a cathodic chamber, and an anodic chamber, as reported in our previous work.[1] An anion exchange membrane (FumasepFAA-3-PK-130) was used to separate the anodic and cathodic chambers, and a Hg/HgO electrode and Ni foam were used as the reference and counter electrodes, respectively. The electrolysis was conducted using a CHI 660e electrochemical workstation equipped with a high current amplifier CHI 680c. The measured potentials after *i*R compensation were rescaled to the reversible hydrogen electrode (RHE) reference by E (versus RHE) = E (versus Hg/HgO) + 0.098 V+0.0591V/pH × pH. For performance studies, 1 M KOH was used as the electrolyte, and it was circulated through the cathodic and anodic chambers using peristaltic pumps at a rate of 30 mL min^{-1}. The flow rate of CO_2 gas through the gas chamber was controlled to be 20 sccm using a digital gas flow controller.

Electrochemical reduction of CO_2 in the membrane electrode assembly (MEA) system. The MEA cell consisted of a titanium anode (cathode) bipolar plate with serpentine flow field, associated nuts, bolts, and insulating kit. An anion-exchange

membrane (AEM, Sustainion X37-50 Grade RT) was sandwiched by the two gas diffusion layer electrodes to separate the chambers. The AEM was activated for at least 24 h in 1 M KOH and rinsed with deionized water before the experiments. The as-prepared gas-diffusion electrode (2.5 cm × 2.5 cm, catalyst loading: 3 mg·cm^{-2}) and an IrO_2/Ti-mesh anode catalysts (3 cm × 3 cm, catalyst loading: 1 mg·cm^{-2}) were employed as the cathode and anode, respectively. The anode was prepared by a dip-coating and thermal decomposition method.[2] During the experiment, 80 sccm humidified CO_2 was supplied to the cathode side, while the anode was circulated with 0.1 M $KHCO_3$ electrolyte at 10 mL min^{-1} flow rate. No *iR* compensation was applied. The gaseous product of electrochemical experiments was collected using a gas bag and analyzed by GC.

Double-layer capacitance (C_{dl}) measurements. The electrochemical active surface area is proportional to C_{dl} value. C_{dl} was determined by measuring the capacitive current associated with double-layer charging from the scan-rate dependence of cyclic voltammogram (CV). The CV ranged from 0.62 V to 0.52 V vs. RHE. The C_{dl} was estimated by plotting the Δj (j_a-j_c) at 0.57 V vs. RHE against the scan rates, in which the j_a and j_c are the anodic and cathodic current density, respectively. The scan rates were 20, 40, 60, 80, 100 and 120 mV s^{-1}.

Product analysis. The gaseous product of electrochemical experiments was collected using a gas bag and analyzed by gas chromatography (GC, HP 4890D), which was equipped with TCD detectors using argon as the carrier gas. The liquid product was

analyzed by ^{1}H NMR (Bruker Avance III 400 HD spectrometer) in deuteroxide with phenol and sodium 2, 2-dimethyl-2-silapentane-5-sulfonate (DSS) as internal standards.

Calculations of Faradaic efficiencies of gaseous and liquid products

Gaseous products:

From the GC peak areas and calibration curves for the TCD detector, we can obtain the V % of gaseous products. Since the flow rate of the outlet was monitored to be constant, the moles of gaseous products can be calculated.

The Faradaic efficiency of gaseous product is:

$$FE = \frac{moles\ of\ product}{Q/nF} \times 100\%$$

(Q: charge (C); F: Faradaic constant (96485 C mol^{-1}); n: the number of electrons required to generate the product)

Liquid products:

After electrolysis, a certain amount of internal standard solution was added to the electrolyte as the internal standard. Because the concentration of internal standard was known, the moles of liquid products can be calculated from integral areas and calibration curves. To accurately integrate the products in NMR analysis, two standards located in different regions were used in NMR analysis. The DSS was the

reference for ethanol, and the phenol was the reference for formate. 400 μL catholyte after the electrocatalytic CO_2 reduction reaction was mixed with 100 μL 6 mM DSS solution, 100 μL 200 mM phenol and 200 μL D_2O, and then analyzed by ^{1}H NMR (Bruker Advance III 400 HD spectrometer).

The Faradaic efficiency of liquid product is:

$$FE = \frac{moles\ of\ product}{Q/nF} \times 100\%$$

(Q: charge (C); F: Faradaic constant (96485 C mol^{-1}); n: the number of electrons required to generate the product)

Computational Method

Spin-polarized density functional theory (DFT) calculations were performed with the Vienna ab initio simulation package (VASP).[3] The projector augmented wave (PAW) pseudopotential for core electrons, and the generalized gradient approximation (GGA) in the form of Perdew−Burke−Ernzerhof (PBE) for the exchange correlation potentials were adopted.[4,5] To avoid interactions between adjacent images, the *c* axis was set to be 25 Å. A cutoff energy of 450 eV was used. The atoms were fully relaxed until the energy convergence reached 0.0001 eV. Van der Waals (vdW) interaction was considered at the DFT-D3 level as proposed by Grimme.

Supplementary Figures

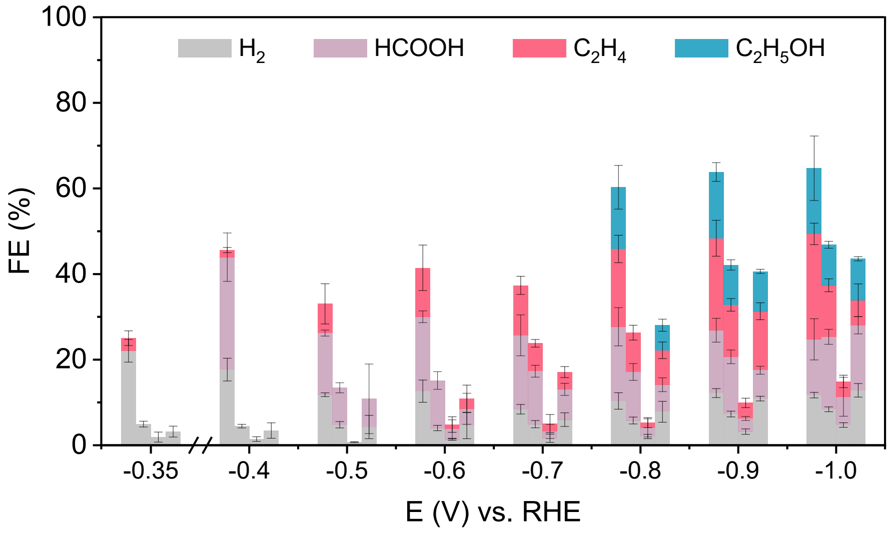

Fig. S1. The FEs of H_2, HCOOH, and C2+ products under different applied potentials over Cu-O, 0.6%Sn-Cu-O, 2.9%Sn-Cu-O, and 4.0%Sn-Cu-O catalysts (in order from left to right).

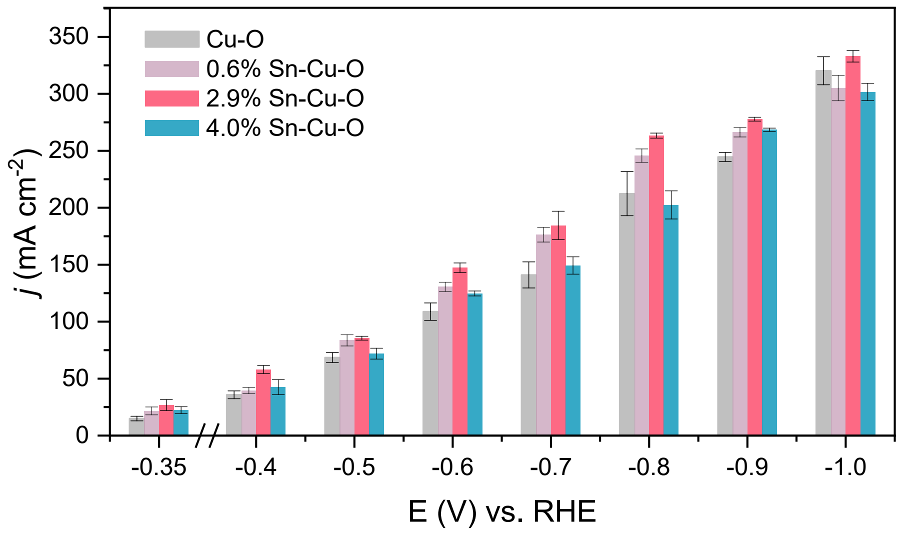

Fig. S2. The total current densities under different applied potentials over Cu-O, 0.6%Sn-Cu-O, 2.9%Sn-Cu-O, and 4.0%Sn-Cu-O catalysts.

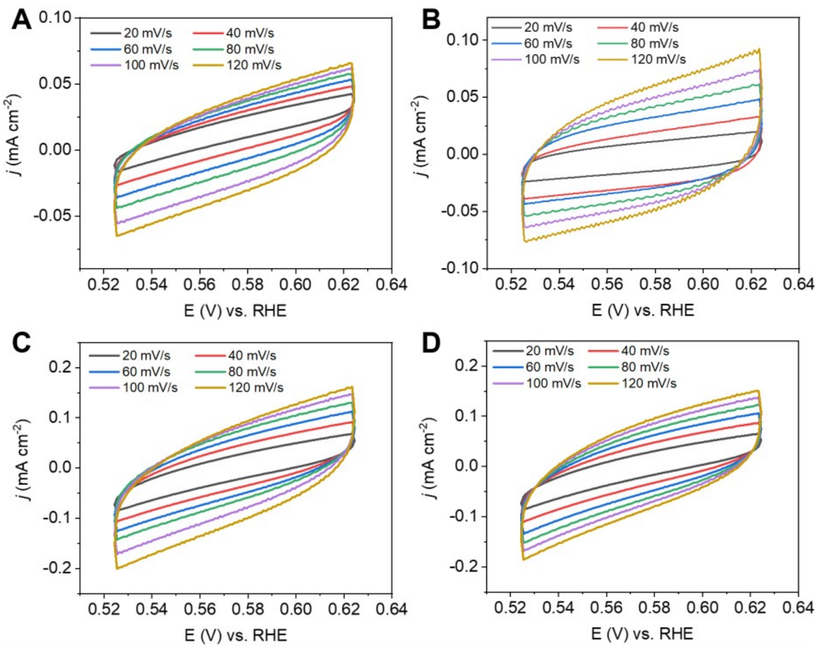

Fig. S3. The cyclic voltammetry at different scan rates (20, 40, 60, 80, 100, 120 mV s^{-1}) over Cu-O (A), 0.6%Sn-Cu-O (B), 2.9%Sn-Cu-O (C), and 4.0%Sn-Cu-O (D).

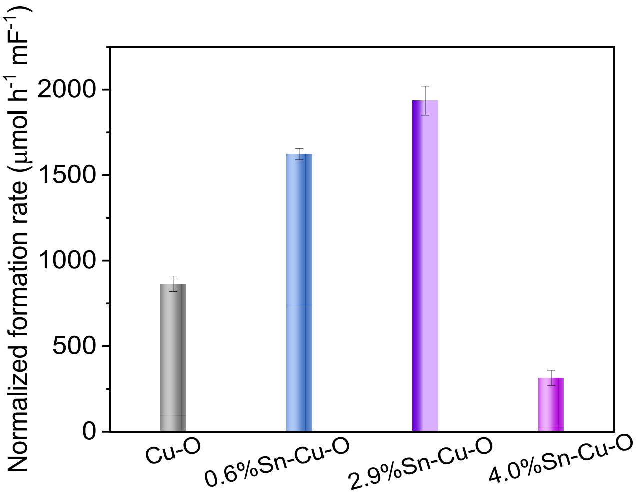

Fig. S4. The ECSA-normalized CO formation rate of Cu-O, 0.6%Sn-Cu-O, 2.9%Sn-Cu-O, and 4.0%Sn-Cu-O catalysts.

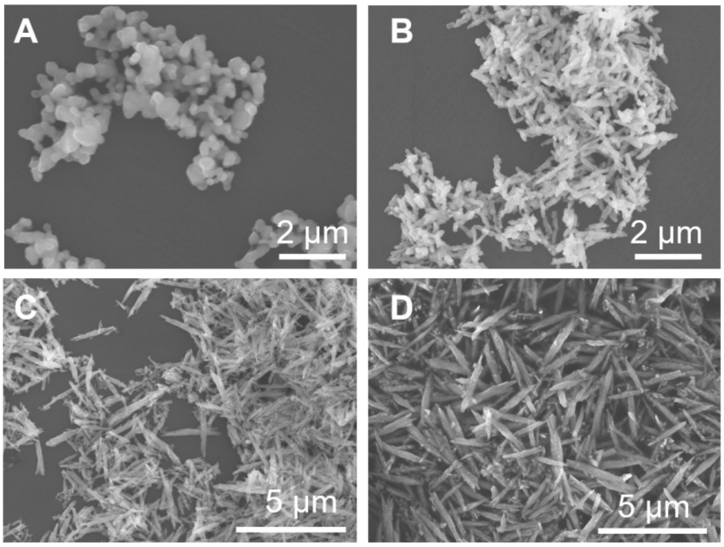

Fig. S5. The SEM images of Cu-O, 0.6%Sn-Cu-O, 2.9%Sn-Cu-O, and 4.0%Sn-Cu-O.

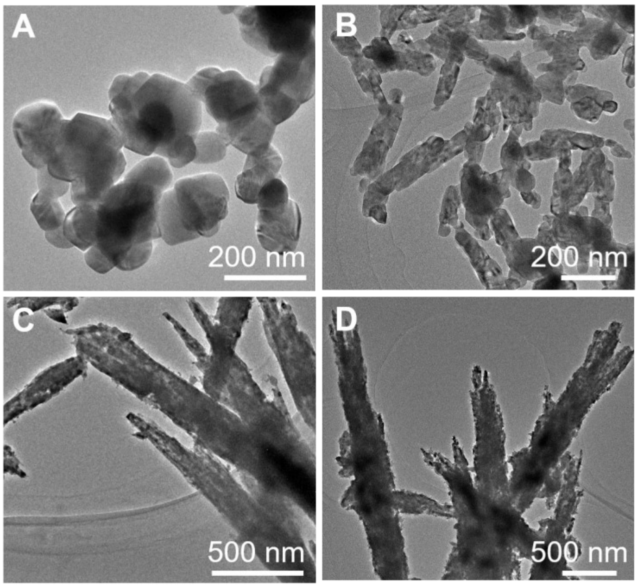

Fig. S6. The TEM images of Cu-O, 0.6%Sn-Cu-O, 2.9%Sn-Cu-O, and 4.0%Sn-Cu-O.

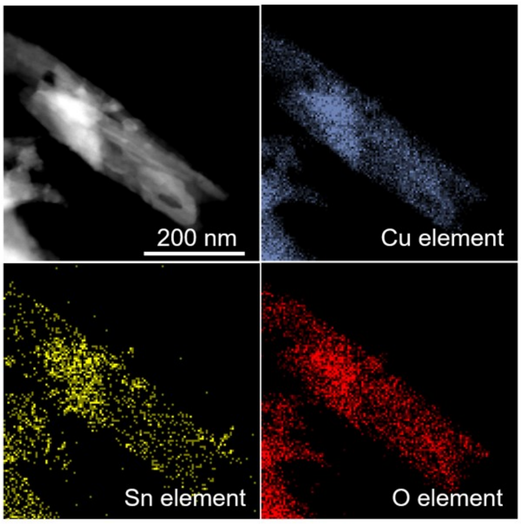

Fig. S7. Energy-dispersive X-ray spectroscopy of 2.9%Sn-Cu-O.

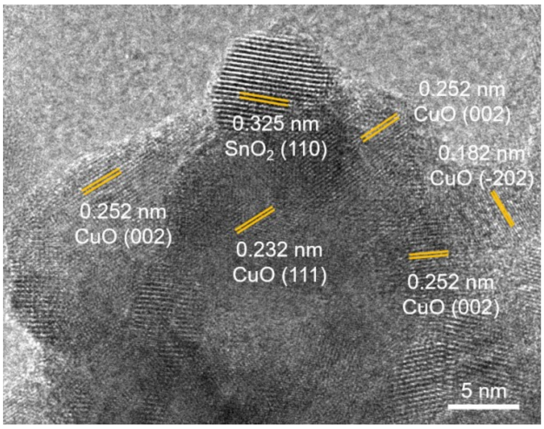

Fig. S8. HR-TEM image of 2.9%Sn-Cu-O.

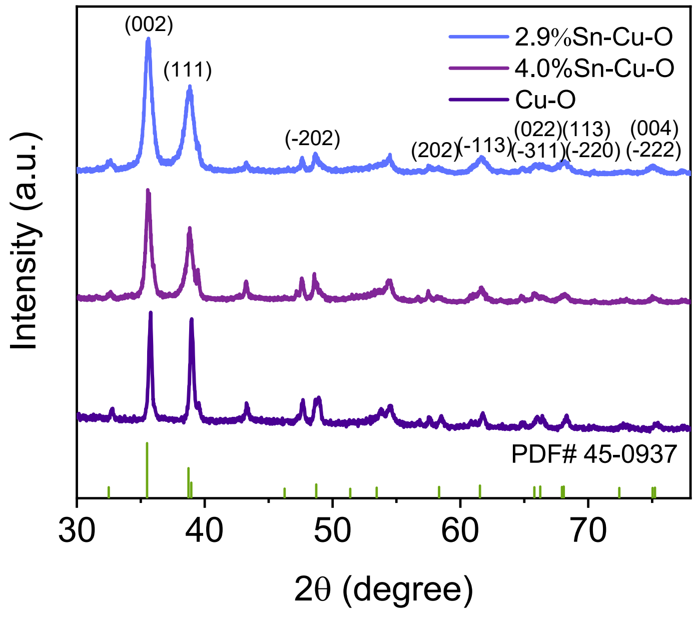

Fig. S9. The XRD patterns of Cu-O, 2.9%Sn-Cu-O, and 4.0%Sn-Cu-O.

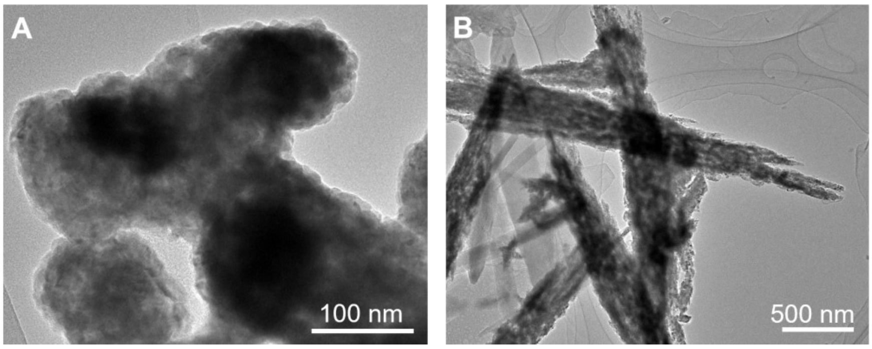

Fig. S10. The TEM images of (A) Cu-O and (B) 4.0%Sn-Cu-O after eCO_2RR.

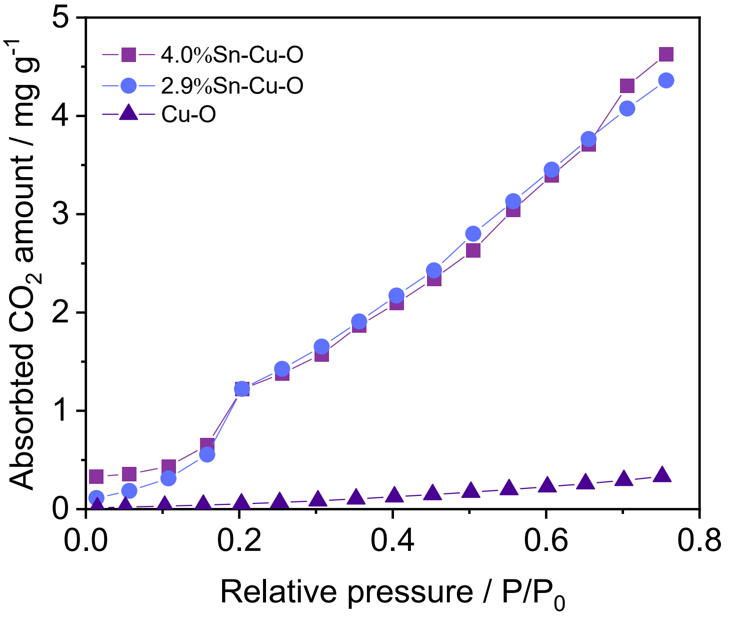

Fig. S11. CO_2 adsorption isotherms at 25 °C of Cu-O, 2.9%Sn-Cu-O, and 4.0%Sn-Cu-O.

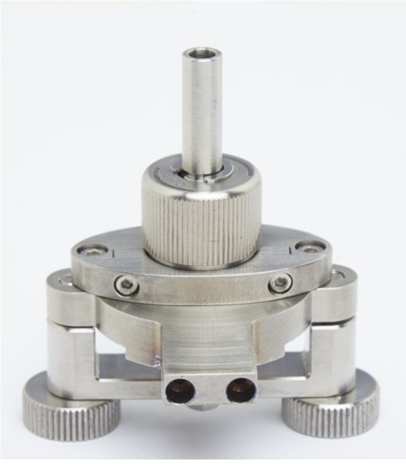

Fig. S12. The quasi-operando XPS cell used in this work.

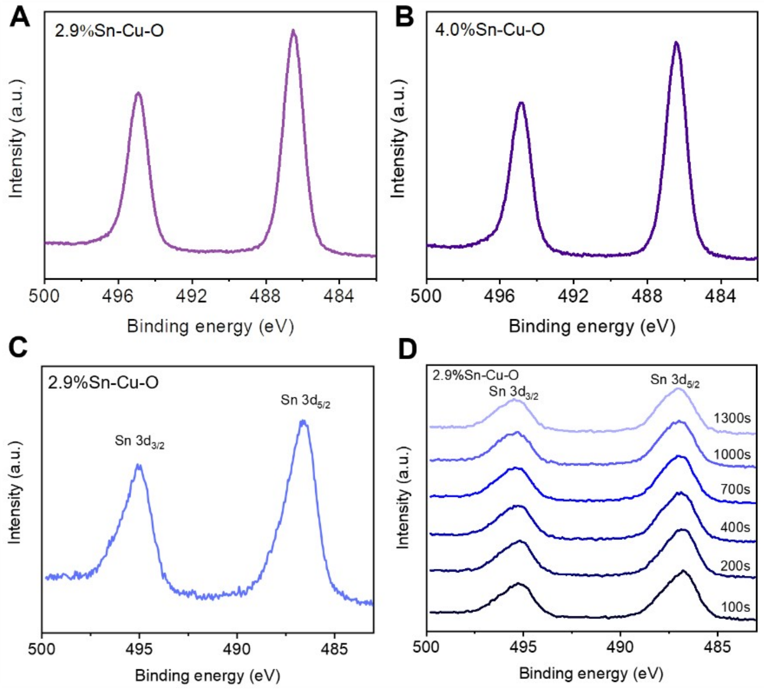

Fig. S13. (A) and (B) XPS spectra of Sn 3d orbits for 2.9%Sn-Cu-O and 4.0%Sn-Cu-O as prepared; (C) The XPS depth profile spectra using an argon cluster beam for 30 min (8 keV energy, 300 atoms size) for 2.9%Sn-Cu-O after eCO_2RR. (D) The XPS depth profile spectra using monatomic argon ions (Ar^+) for different times for 2.9%Sn-Cu-O after eCO_2RR (the etching depth was estimated to be 30 to 380 nm while the surface oxide layer formed by air oxidation is usually < 5nm) [6].

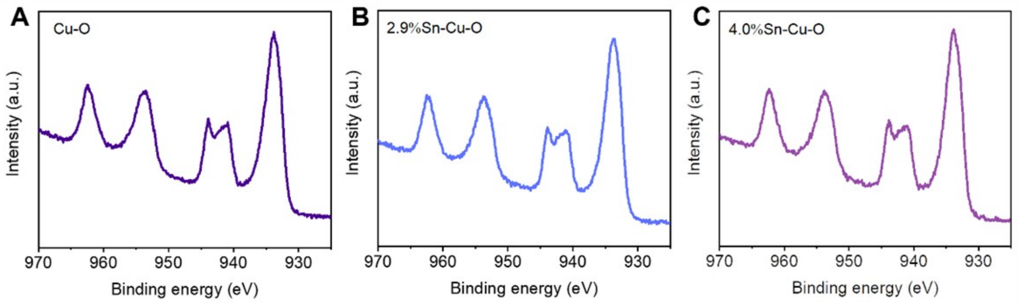

Fig. S14. XPS spectra of Cu 2p orbits for Cu-O, 2.9%Sn-Cu-O, and 4.0%Sn-Cu-O.

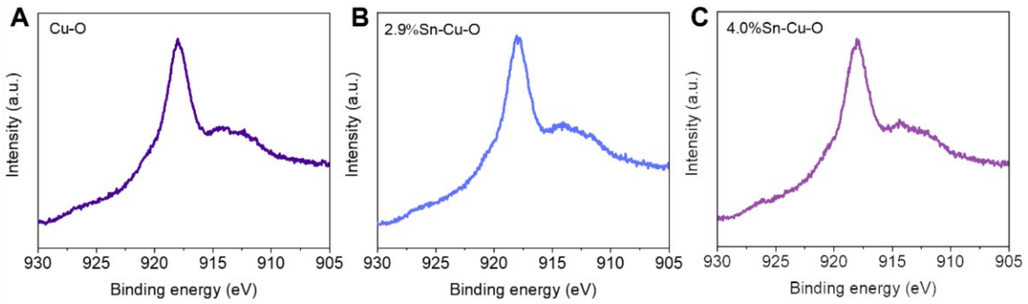

Fig. S15. Cu $L_3M_{45}M_{45}$ Auger spectroscopy for Cu-O, 2.9%Sn-Cu-O, and 4.0%Sn-Cu-O.

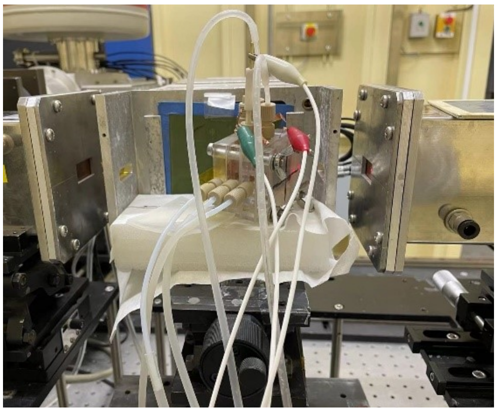

Fig. S16. Operando electrochemical cell for XAS test.

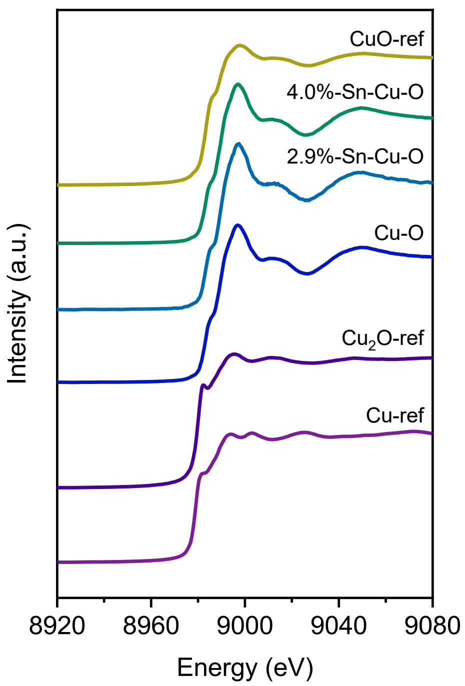

Fig. S17. XANES spectra at the Cu K-edge of Cu-O, 2.9%Sn-Cu-O, and 4.0%Sn-Cu-O.

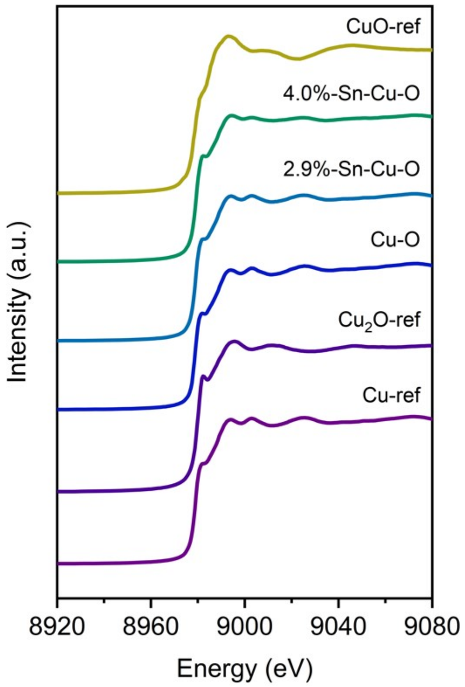

Fig. S18. Operando XANES spectra at the Cu K-edge of Cu-O, 2.9%Sn-Cu-O, and 4.0%Sn-Cu-O under an appiled potential of -0.6 V vs. RHE during eCO_2RR.

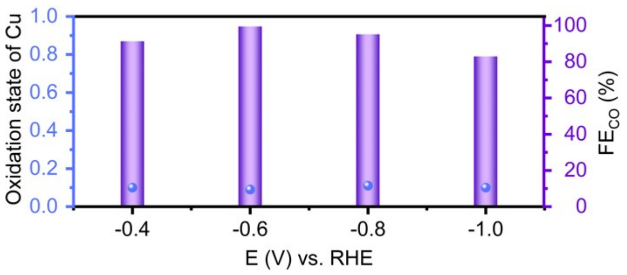

Fig. S19. The oxidation state of Cu and FE_{CO} over 2.9%Sn-Cu-O at different cathodic potential.

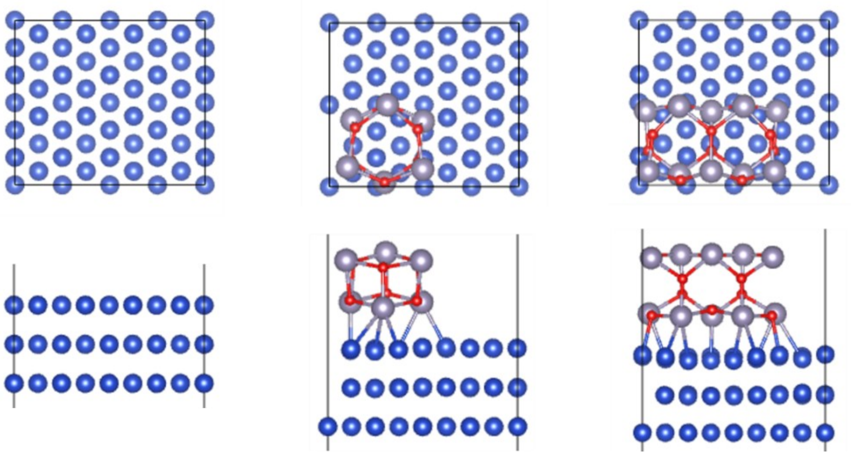

Fig. S20. Well optimized substrates of Cu(111), Sn_{less}-Cu(111), and Sn_{more}-Cu(111).

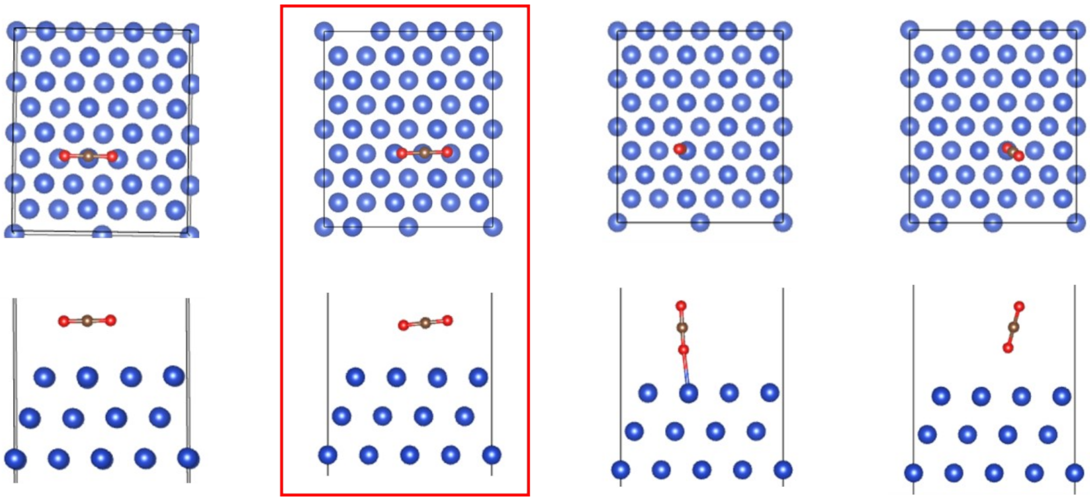

Fig. S21. Comparison of different configurations of adsorbed CO_2 on Cu (111) (The one circled in red is the optimal conformation).

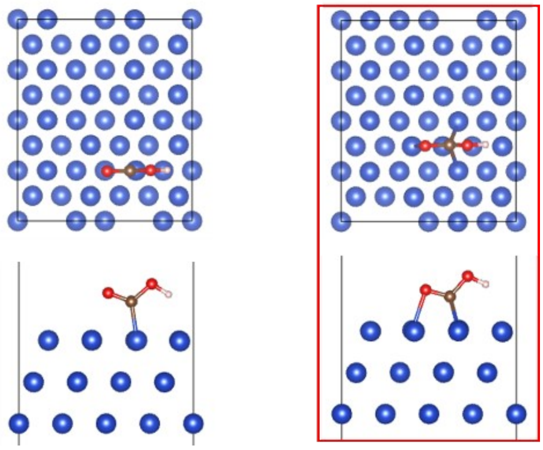

Fig. S22. Comparison different configurations of adsorbed COOH on Cu (111) (The one circled in red is the optimal conformation).

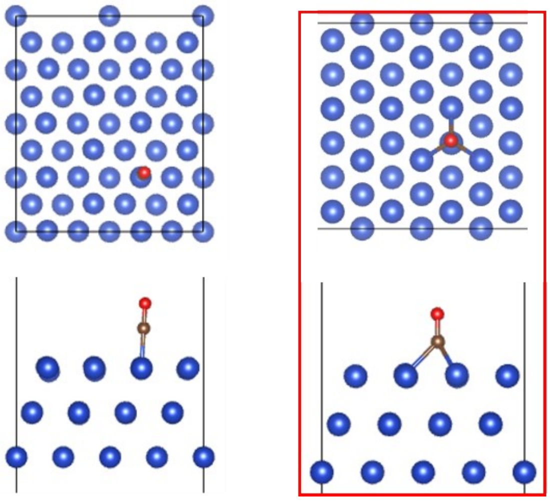

Fig. S23. Comparison different configurations of adsorbed CO on Cu (111) (The one circled in red is the optimal conformation).

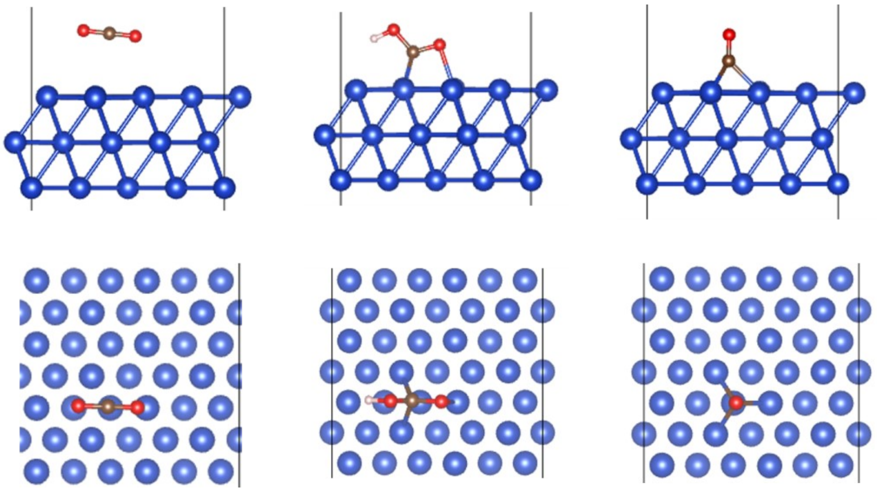

Fig. S24. Final adsorption configurations on Cu (111).

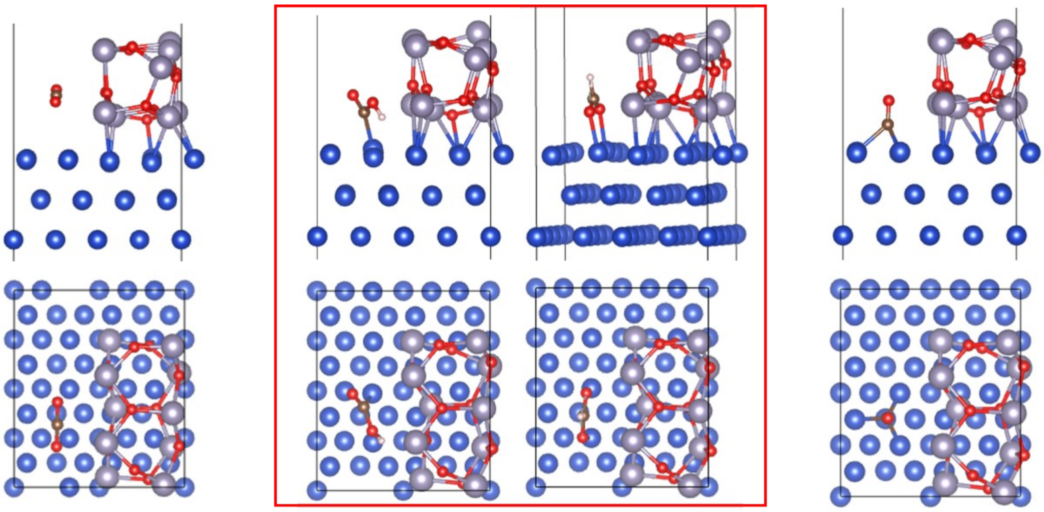

Fig. S25. Final adsorption configurations on Sn_{more}-Cu(111).

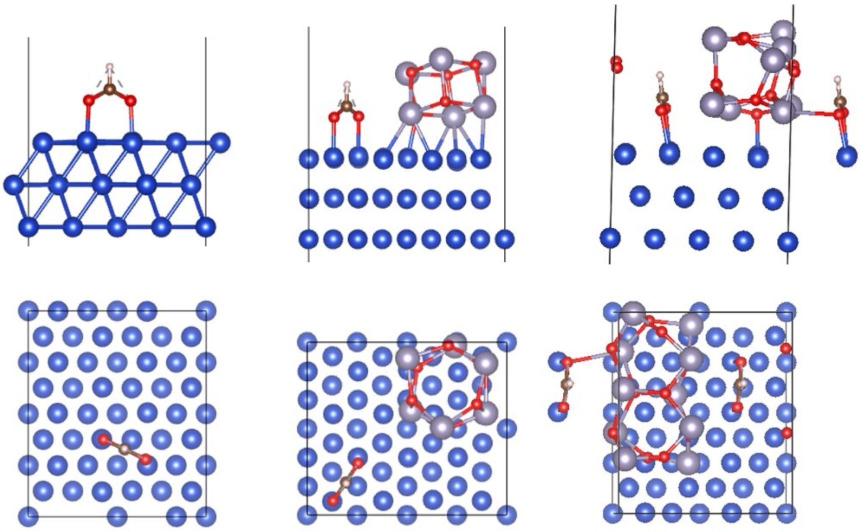

Fig. S26. Final adsorption configurations of *OCHO on Cu(111), Sn_{less}-Cu(111), and Sn_{more}-Cu(111).

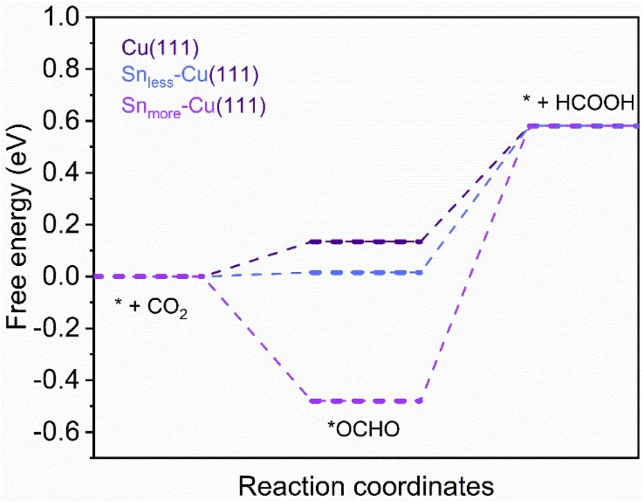

Fig. S27. Gibbs free-energy diagrams for eCO_2RR to HCOOH on different simulated models.

Supplementary Tables

Table S1. Recently reported Cu-Sn catalysts for eCO_2RR.

Catalysis	Applied potential (V vs. RHE)	FE_{CO} (%)	FE_{HCOOH} (%)	Current density (mA cm^{-2})	Reference
7/0.8 Cu/SnO_2	-0.7 V	93	-	-	7
Cu@Sn nanocones	-1.1 V	-	90.4%	57.7	8
Cu-Sn dendrite	-0.7 V	~90	-	11.5 (-1.1 V)	9
Cu-Sn foam	-0.7	94	-	4.7 (-0.8 V)	10
Cu-Sn nanoparticles	-0.95	-	92	9	11
Sn/Cu-nanofiber	-1.0	80	-	53	12
OD-Cu-Sn	-0.6	90	-	1.0	13
Cu-Sn electrodeposition	-1.1	-	81	13	14
$Cu_{70}Sn_{30}$ bulk alloy	-1.0	-	88.8	92	15
$Cu_{97}Sn_3$ surface alloy	-0.7	98	-	78	16

Table S2. Recently reported catalysts for eCO_2RR to CO in flow-cells.

Catalysis	Applied potential (V vs. RHE)	FE_{CO} (%)	Current density of CO (mA cm^{-2})	Electrolyte	Reference
2.9%Sn-Cu-O	-0.6	99.5	145	1 M KOH	This work
2.9%Sn-Cu-O	0.9	95.4	265	1 M KOH	This work
Au	-0.7 V	~110	~90	1 M KOH	17
CoPc	-0.65	94	70.5	1 M KOH	18
CoPc-N	-0.66	94	31	1 M KOH	19
Ni-N-C	-0.7	90	~85	1M $KHCO_3$	20
Ni-N-C	-0.8	97	~110	0.5 M $KHCO_3$	21
Fe-N-C	-0.45	~90	94	0.5 M $KHCO_3$	22
Ni-N/PCFM	-0.7	~80	~115	0.5 M $KHCO_3$	23
Ag/PTFE	-1.0	~90	~160	1M $KHCO_3$	24
Ag/PTFE	-0.7	90	~150	1 M KOH	24
Ag/MPL-3C	-0.7	98.5	98.5	0.1 M $KHCO_3$	25
CoSA/HCNFs	-0.6	~70	~100	1M $KHCO_3$	26
CoPc@Fe-N-C	-0.55	>90%	~100	0.5 M KOH	27

Table S3. The correction of zero point energy and entropy effect of the adsorbed and gaseous species.

	ZPE (eV)	TS (eV)
H_2O	0.57	0.67
H_2	0.27	0.4
CO_2	0.3067	0.6671
CO	0.1316	0.6154
HCOOH	0.8791	0.7762

References

1 C. Chen, X. Yan, S. Liu, Y. Wu, Q. Wan, X. Sun, Q. Zhu, H. Liu, J. Ma, L. Zheng, H. Wu, B. Han, *Angew. Chem. Int. Ed.* 2020, **59**, 16459-16464.

2 W. Luc, J. Rosen, F. Jiao, *Catal. Today* 2017, **288**, 79-84.

3 G. F. l. Kresse, J., *Comput. Mater. Sci.* 1996, **6**, 15-50.

4 P. E. Blochl, *Phys. Rev., B Condens. Matter* 1994, **50**, 17953-17979.

5 J. P. B. Perdew, K.; Ernzerhof, M., *Phys. Rev. Lett.* 1996, **77**, 3865-3868.

6 W. Liu, P. Zhai, A. Li, B. Wei, K. Si, Y. Wei, X. Wang, G. Zhu, Q. Chen, X. Gu, R. Zhang, W. Zhou and Y. Gong, *Nat. Commun.* 2022, 13, 1877.

7 Q. Li, J. Fu, W. Zhu, Z. Chen, B. Shen, L. Wu, Z. Xi, T. Wang, G. Lu, J. J. Zhu, S. Sun, *J. Am. Chem. Soc.* 2017, **139**, 4290-4293.

8 C. Chen, Y. Pang, F. Zhang, J. Zhong, B. Zhang, Z. Cheng, *J. Mater. Chem. A* 2018, **6**, 19621 – 19630.

9 W. Ju, J. Zeng, K. Bejtka, H. Ma, D. Rentsch, M. Castellino, A. Sacco, C. F. Pirri, C. Battaglia, *ACS Appl. Energy Mater.* 2018, **2**, 867-872.

10 J. Zeng, K. Bejtka, W. Ju, M. Castellino, A. Chiodoni, A. Sacco, M. A. Farkhondehfal, S. Hernández, D. Rentsch, C. Battaglia, C. F. Pirri, *Appl. Catal. B: Environ*. 2018, **236**, 475-482.

11 X. Zheng, Y. Ji, J. Tang, J. Wang, B. Liu, H.-G. Steinrück, K. Lim, Y. Li, M. F. Toney, K. Chan, Y. Cui, *Nat. Catal.* 2018, **2**, 55-61.

12 X. Jiang, X. Wang, Z. Liu, Q. Wang, X. Xiao, H. Pan, M. Li, J. Wang, Y. Shao, Z. Peng, *Appl. Catal. B: Environ.* 2019, **259**, 118040.

13 W. Ju, F. Jiang, H. Ma, Z. Pan, Y. B. Zhao, F. Pagani, D. Rentsch, J. Wang, C. Battaglia, *Adv. Energy Mater.* 2019, **9**, 1901514.

14 S. Sarfraz, A. T. Garcia-Esparza, A. Jedidi, L. Cavallo, K. Takanabe, *ACS Catal.* 2016, **6**, 2842-2851.

15 M. Li, X. Tian, S. Garg, T. E. Rufford, P. Zhao, Y. Wu, A. J. Yago, L. Ge, V. Rudolph, G. Wang, *ACS Appl. Mater. Inter.* 2020, **12**, 22760-22770.

16 C. J. Yoo, W. J. Dong, J. Y. Park, J. W. Lim, S. Kim, K. S. Choi, F. O. Odongo Ngome, S.-Y. Choi, J.-L. Lee, *ACS Appl. Energy Mater.* 2020, **3**, 4466-4473.

17 S. Verma, Y. Hamasaki, C. Kim, W. Huang, S. Lu, H.-R. M. Jhong, A. A. Gewirth, T. Fujigaya, N. Nakashima, P. J. A. Kenis, *ACS Energy Lett.* 2017, **3**, 193-198.

18 M. Wang, K. Torbensen, D. Salvatore, S. Ren, D. Joulie, F. Dumoulin, D. Mendoza, B. Lassalle-Kaiser, U. Isci, C. P. Berlinguette, M. Robert, *Nat. Commun.* 2019, **10**, 3602.

19 X. Lu, Y. Wu, X. Yuan, L. Huang, Z. Wu, J. Xuan, Y. Wang, H. Wang, *ACS Energy Lett.* 2018, **3**, 2527-2532.

20 T. Möller, W. Ju, A. Bagger, X. L. Wang, F. Luo, T. Ngo Thanh, A. S. Varela, J. Rossmeisl, P. Strasser, *Energy Environ. Sci.* 2019, **12**, 640−647.

21 T. Zhang, L. Lin, Z. Li, X. He, S. Xiao, V. N. Shanov, J. Wu, *ACS Appl. Energy Mater.* 2020, **3**, 1617-1626.

22 J. Gu, C.-S. Hsu, L. Bai, H. M. Chen, X. Hu, *Science* 2019, **364**, 1091-1094.

23 H. Yang, Q. Lin, C. Zhang, X. Yu, Z. Cheng, G. Li, Q. Hu, X. Ren, Q. Zhang, J. Liu, C. He, *Nat. Commun.* 2020, **11**, 593.

24 C.-T. Dinh, F. P. García de Arquer, D. Sinton, E. H. Sargent, *ACS Energy Lett.* 2018, **3**, 2835-2840.

25 R. Wang, H. Haspel, A. Pustovarenko, A. Dikhtiarenko, A. Russkikh, G. Shterk, D. Osadchii, S. Ould-Chikh, M. Ma, W. A. Smith, K. Takanabe, F. Kapteijn, J. Gascon, *ACS Energy Lett.* 2019, **4**, 2024-2031.

26 H. Yang, Q. Lin, Y. Wu, G. Li, Q. Hu, X. Chai, X. Ren, Q. Zhang, J. Liu, C. He, *Nano Energy* 2020, **70**, 104454.

27 L. Lin, H. Li, C. Yan, H. Li, R. Si, M. Li, J. Xiao, G. Wang, X. Bao, *Adv. Mater.* 2019, **31**, 1903470.
